# Management of Pathological Dental Attrition in Prader–Willi Syndrome: A Case Report Using the Personalized Radboud Strategy

**DOI:** 10.1111/scd.70149

**Published:** 2026-02-11

**Authors:** Hoda Tayebi‐Hillali, Pablo Fernández Alonso, Berta Rivas‐Mundiña, Mercedes Outumuro Rial, Márcio Diniz‐Freitas, Javier Fernández Feijoo

**Affiliations:** ^1^ Medical‐Surgical Dentistry Research Group (OMEQUI) Health Research Institute of Santiago de Compostela (IDIS) University of Santiago de Compostela (USC) Santiago De Compostela Spain

**Keywords:** behavioral management, case report, dental wear, general anesthesia, Prader–Willi syndrome, removable prosthesis

## Abstract

Prader–Willi syndrome (PWS) is a rare genetic disorder characterized by obesity, hypotonia, intellectual disability, and behavioral disturbances that complicate dental management. Parafunctional habits such as bruxism often lead to severe tooth wear, while cooperation and anesthesia represent additional challenges. A 34‐year‐old woman with genetically confirmed PWS presented with generalized dental wear, poor oral hygiene, and multiple carious lesions. Preventive and splint therapies were initially proposed but not feasible. Two years later, she returned with pain due to pulp exposure. Because of limited cooperation and comorbidities, dental treatment under general anesthesia was planned in two sessions, including molar extractions and multiple root canal treatments. Complete acrylic dentures with metal reinforcement were fabricated, restoring vertical dimension, improving esthetics, and serving as protective splints. Caregivers were instructed on hygiene, and annual follow‐up was established. After six years, bone atrophy and further wear were noted, but the patient continued using relined prostheses without sedation. This case demonstrates that a resolutive, interdisciplinary approach can successfully manage complex dental problems in PWS. General anesthesia minimized the number of interventions while ensuring comprehensive care. Reinforced acrylic dentures provided a functional, aesthetic, and cost‐effective solution, despite the progressive nature of dental wear and bone loss.

## 1 Introduction

Prader‐Willi syndrome (PWS) is a rare genetic disorder resulting from a deletion in the 15q11‐q13 region of chromosome 15 [1]. First described in 1956 by Swiss pediatricians Andrea Prader and Heinrich Willi, along with internist Alexis Labhart, PWS is characterized by a complex phenotype affecting multiple systems [2]. The most prominent clinical features include neonatal hypotonia, intellectual disability, language deficits, hyperphagia leading to severe obesity, endocrine dysfunctions, and behavioral abnormalities like chronic skin‐picking, hyperphagia or impulsivity, and mood swings [1, 3, 4, 5]. Additionally, individuals with PWS frequently present with epilepsy [6]. The syndrome is not influenced by sex, race, or socioeconomic status [2]. Its estimated global prevalence is approximately 1 in 25 000 live births, with around 3000 confirmed cases in Spain [7, 8, 9].

Individuals with PWS exhibit a wide spectrum of oral and dental anomalies, including caries [10, 11], enamel hypoplasia [11, 12], periodontal disease [13], acid erosion [14], rampant caries [12, 15], oral microsomia [11, 12], dental malocclusions [16], oral candidiasis [11, 17], erythematous lesions of the oral mucosa [18], excessive biofilm accumulation [11, 12, 18], gingivitis, angular cheilitis [19], dentoalveolar abscesses, lingual hypotonia, mouth breathing, delayed tooth eruption, and dental wear [12, 20, 21, 22, 23]. Dental wear could be attributed to pathological attrition; however, studies confirm that in PWS, it may result from attrition, erosion, or a combination of both processes [24]. Erosion is closely associated with gastroesophageal reflux and the consumption of acidic foods and beverages [25].

Additionally, PWS patients commonly exhibit both quantitative (hyposalivation) and qualitative (acidic pH and altered protein content) salivary dysfunction, which compromises the protective role of saliva against enamel demineralization [22, 26, 27]. This condition may be further exacerbated by the use of xerostomic medications, which are often prescribed to aid in behavioral management [28]. Moreover, behavioral disturbances in individuals with PWS can contribute to self‐inflicted oral lesions [18].

In 2017, a European Consensus document on the management of dental wear was published, stating that treatment should depend on the severity of the lesions and the patient's demands, and that restorative procedures should be postponed as much as possible while remaining minimally invasive [29]. A few months later, two of the authors of that consensus published a series of explicit recommendations based on their clinical experience at Radboud University (Netherlands), which they termed the “Radboud philosophy” and summarized in five key points: a) Restorative treatment is not always indicated, even in cases of severe dental wear. b) In asymptomatic cases, counseling and follow‐up are the best approach. c) In cases of increased vertical dimension, especially in young individuals, the preferred strategy is adhesive and minimally invasive restorations. d) Clinical evidence supporting the success of direct composite resin restorations is limited to five years. e) The informed consent process must specify the available treatment options and potential complications, highlighting that restorations may have a limited lifespan due to bruxism and erosion [30].

Both the European Consensus and the Radboud philosophy have limitations when applied to PWS patients, which are particularly evident in this clinical case. The treatment demand of a patient with an intellectual disability may become the demand of their legal guardians, which may sometimes be primarily driven by aesthetic concerns. Symptomatology is also an unreliable criterion, as many PWS patients have altered sensory perception with a notably high pain threshold [31].

## 2 Case Report

This case report has been written following the CARE guidelines [32] (Supplementary 1).

A 34‐year‐old woman diagnosed with PWS (genetically confirmed) attended a consultation accompanied by her mother, who sought treatment for her generalized dental wear. The patient had grade II obesity, sleep apnea, generalized muscle hypotonia, acromicria, compulsive eating behavior, intellectual disability, and a characteristic behavioral phenotype, including frequent mood swings and episodes of heteroaggressiveness. She was under pharmacological treatment with risperidone, paroxetine, and topiramate.

**FIGURE 1:**
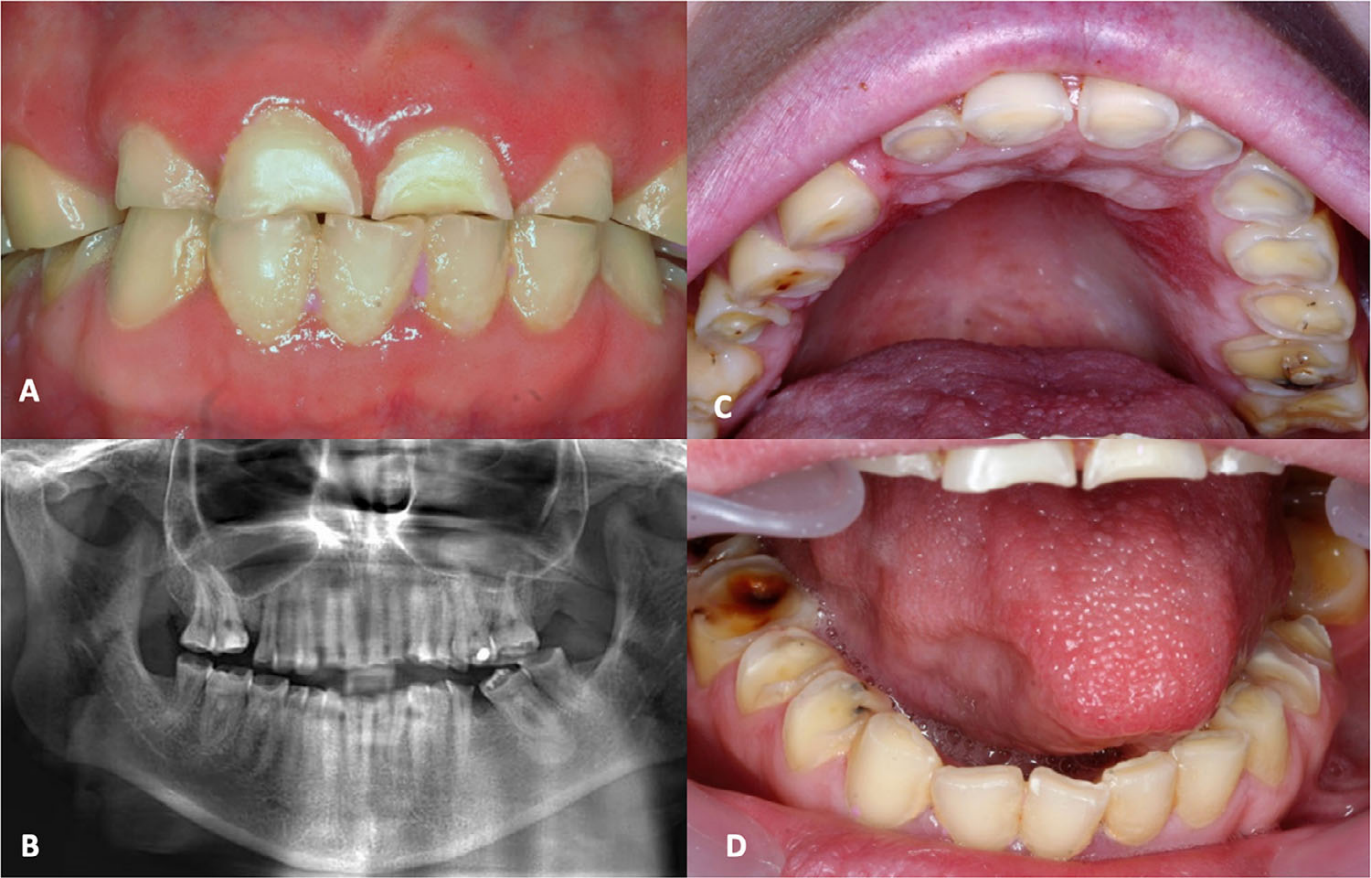
Clinical and radiographic findings in the initial examination. (A) Intraoral view of dental wear in occlusion. (B) Panoramic radiography showing dental wear and missing teeth. (C) Dental wear in the maxillary arch. (D) Dental wear in the mandibular arch.

During the first visit, she demonstrated a reasonable level of comprehension and good interaction with the clinician, although she was resistant to some verbal instructions. Oral examination confirmed labial (poor seal) and lingual hypotonia, mouth breathing, atypical swallowing, Class II malocclusion, and poor oral hygiene. Multiple carious lesions were identified (teeth 17, 18, 26, 28, 37, 38, 46, 47, and 48), as well as missing teeth (16, 27, and 36). Notably, she exhibited generalized dental wear, which was attributed to attrition due to bruxism (Figure 1).

The dental wear was asymptomatic; therefore, following conventional criteria, it was decided to postpone any restorative procedures, provide preventive recommendations (oral hygiene and dietary counseling), and propose the fabrication of protective splints, an option ultimately deemed unfeasible by the family. Two years later, the patient returned with self‐perceived pain, which was attributed to pulp exposure. Given the suspicion of pain due to pulp chamber exposure, root canal treatment was proposed, which is a procedure not mentioned in these publications and only documented in the literature for a single PWS patient (a central incisor) [33].

**FIGURE 2:**
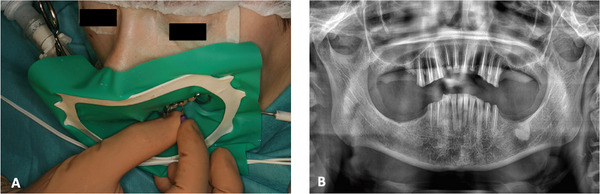
(A) Details of the endodontic procedure performed under general anesthesia, and (B) The final panoramic radiograph obtained after completing the extractions, root canal treatments, and restorations.

After discussing the case in a clinical session with anesthesiologists, two hospital‐based treatment sessions under general anesthesia were planned. During the first intervention, extractions of the first and fourth quadrant molars (17, 18, 46, 47, and 48) were performed, and root canal treatments were carried out on teeth 11, 12, 13, 14, 15, 21, 22, 23, 24, and 25. In the second session, extractions of the second and third quadrant molars (26, 28, 37, and 38) were performed, along with root canal treatments on teeth 34, 35, 44, and 45. Root canal procedures involved manual pre‐instrumentation with K‐files, rotary instrumentation using the ProTaper system, and canal obturation with the warm gutta‐percha technique of the Thermafil system (Dentsply Sirona, Charlotte, NC, USA) (Figure 2). Following obturation, impressions of both arches were taken using silicone, along with a registration of the intermaxillary relationship.

Two complete acrylic dentures (upper and lower) were designed, incorporating a metal reinforcement in the area of maximum occlusal contact to prevent wear caused by bruxism. The patient tolerated a wax trial and the fitting of the definitive dentures in the dental chair. The prostheses were designed as protective splints against dental attrition while also serving functional and aesthetic purposes. In the anterior region, they restored the lost vertical dimension, and the buccal surfaces of the acrylic teeth were extended beyond the gingival sulcus of the remaining natural teeth to enhance the esthetics of the smile.

**FIGURE 3:**
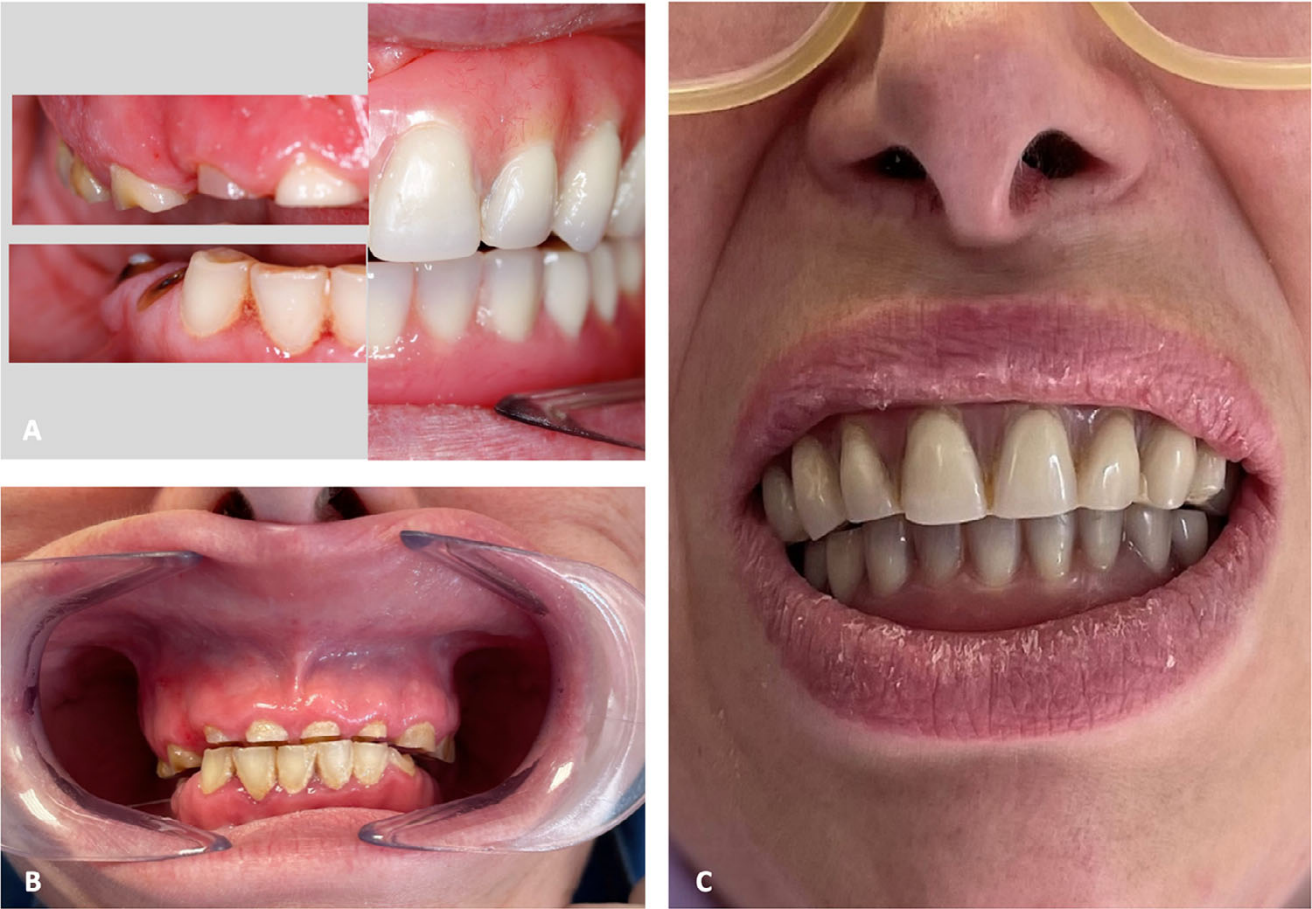
After 6 years of follow‐up (A) Comparison between remaining natural teeth and prosthetic teeth. (B) Progression of dental wear is observed. (C) The rehabilitation remains both functionally and aesthetically acceptable.

**FIGURE 4:**
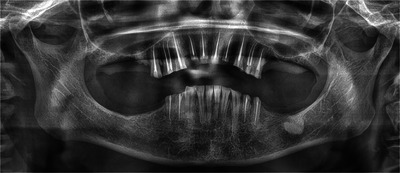
Six‐year follow‐up radiograph.

The patient and her family were instructed in oral hygiene techniques and supervised brushing. The use of fluoride and chlorhexidine mouthwashes was prescribed according to a structured regimen, and annual follow‐up visits were scheduled. After six years of follow‐up, substantial bone atrophy was observed, particularly in the edentulous areas, along with progressive loss of the coronal structures of the remaining teeth, confirming that dental wear was not exclusively due to attrition from bruxism (Figures 3 and 4). The patient continued to use the dentures regularly, which were relined in the dental office without the need for pharmacological sedation.

## 3 Discussion

This case underscores the complexities of managing dental care in patients with PWS, particularly concerning behavioral challenges, bruxism‐induced dental wear, and the necessity for interdisciplinary treatment strategies.

The rehabilitative approach focused on restoring both function and esthetics while providing a protective mechanism against further attrition. The prostheses also played a crucial role in restoring the lost vertical dimension and enhancing facial esthetics. Despite initial concerns regarding patient compliance, the dentures were well‐tolerated, and their regular use contributed to the long‐term success of the treatment. Longitudinal follow‐up over six years revealed substantial bone atrophy in edentulous areas and progressive loss of the coronal structures of the remaining teeth. This observation suggests that factors beyond attrition from bruxism contributed to dental wear, potentially including occlusal dysfunctions and systemic metabolic alterations associated with PWS. The ability to reline the dentures without pharmacological sedation highlights the adaptability of this rehabilitative approach and its feasibility in patients with special needs.

A key factor in designing the therapeutic strategy was the patient's limited cooperation. Sedation for dental treatments in patients with PWS has been contraindicated due to obesity, hypotonia, obstructive sleep apnea, and respiratory limitations [34]. General anesthesia can also be complicated by morbid obesity, airway management difficulties, the risk of perioperative respiratory failure, disturbances in central respiratory control and temperature regulation, and, less frequently, cardiac abnormalities, aggressive behavior, and glucose intolerance [34, 35]. After the mandatory evaluation by anesthesiology specialists, it was decided to plan the dental treatment in two sessions under general anesthesia, each not exceeding three hours in duration, as the risk of complications generally increases the longer interventions are prolonged [36].

This anesthetic approach required the extraction of the posterior teeth due to the impossibility of performing root canal treatments on the molars. In this regard, in patients with intellectual disabilities, the prognosis for root canal treatments performed under general anesthesia may even be better than those performed with the patient awake [37].

Numerous materials have been tested for direct and indirect restorations in patients with dental wear, including metal‐ceramic crowns, gold, lithium disilicate ceramics, zirconia, polymer‐infiltrated ceramic networks, and composite resins, but no restoration technique has demonstrated significantly superior results over others [38]. The Radboud philosophy proposes performing restorative treatment with direct composite resins [30]. Although it has been suggested that severe dental wear often requires prosthodontic rehabilitations in young adults, only two cases of prosthetic rehabilitation in patients with PWS have been found in the literature [38, 39]. In one case, sedation sessions were combined with one general anesthesia session to perform a root canal and fabricate a fixed prosthesis involving the upper central incisors [38]. In the other, crown lengthening and full rehabilitation with a ceramic prosthesis were performed using parenteral sedation [39]. In this patient, the extraction of the molars required proposing a prosthetic rehabilitation with an uncertain prognosis. Therefore, we opted for an acrylic removable prosthesis with a metal reinforcement in the occlusal area to protect the remaining dental structures, thereby minimizing the number of sessions required under general anesthesia. After six years of follow‐up, this approach has proven to be a functional, aesthetically acceptable, and cost‐effective solution.

This case reinforces the importance of an individualized, interdisciplinary strategy when managing dental care in PWS patients. Collaboration between dentists, anesthesiologists, and caregivers is essential to optimize outcomes while minimizing risks. Future studies should explore alternative prosthetic materials and strategies to better address the long‐term consequences of dental wear and bone atrophy in this patient population.

## 4 Conclusion

This clinical case of a patient with PWS, who was minimally cooperative and presented with odontalgia due to severe dental wear, was successfully managed through a resolutive approach performed under general anesthesia, which has no precedents in the literature, with the goal of minimizing the need for future treatment sessions.

## Funding

The authors declare that no funding or external support was received for the preparation of this case report.

## Ethics Statement

The authors have nothing to report

## Consent

Written informed consent was obtained from the patient's legal guardian for the diagnostic procedures, treatment, and for the publication of this case report, including all accompanying clinical data and images.

## Conflicts of Interest

The authors declare no conflicts of interest related to this case report.

## Supporting information




**Supplementary 1**: CARE Checklist.
