# Supplementary material for: Management of Pathological Dental Attrition in Prader–Willi Syndrome: A Case Report Using the Personalized Radboud Strategy
Source: Spec Care Dentist. 2026 Feb 11;46(1):e70149. doi: 10.1111/scd.70149 (PMC12892171; doi:10.1111/scd.70149)
Supplement: Supplementary file 1 — Supplementary 1: CARE Checklist. [file SCD-46-0-s001.pdf]

CARE case report guidelines

CARE Checklist of information to include when writing a case report

(cc) BY-NC-ND

Topic	Item	Checklist item description	Reported on Line
Title	1	The diagnosis or intervention of primary focus followed by the words “case report”	1-2
Key Words	2	2 to 5 key words that identify diagnoses or interventions in this case report, including "case report"	27-28
Abstract (no references)	3a	Introduction: What is unique about this case and what does it add to the scientific literature?	6-9
	3b	Main symptoms and/or important clinical findings	10-19
	3c	The main diagnoses, therapeutic interventions, and outcomes	10-19
	3d	Conclusion—What is the main “take-away” lesson(s) from this case?	20-25
Introduction	4	One or two paragraphs summarizing why this case is unique (**may include references**)	32-78
Patient Information	5a	De-identified patient specific information.	85-96
	5b	Primary concerns and symptoms of the patient.	98-104
	5c	Medical, family, and psycho-social history including relevant genetic information	85-90
	5d	Relevant past interventions with outcomes	98-104
Clinical Findings	6	Describe significant physical examination (PE) and important clinical findings.	87-94
Timeline	7	Historical and current information from this episode of care organized as a timeline	96-105
Diagnostic Assessment	8a	Diagnostic testing (such as PE, laboratory testing, imaging, surveys).	91-96
	8b	Diagnostic challenges (such as access to testing, financial, or cultural)	91-92
	8c	Diagnosis (including other diagnoses considered)	91-104
	8d	Prognosis (such as staging in oncology) where applicable	
Therapeutic Intervention	9a	Types of therapeutic intervention (such as pharmacologic, surgical, preventive, self-care)	106-123
	9b	Administration of therapeutic intervention (such as dosage, strength, duration)	106-123
	9c	Changes in therapeutic intervention (with rationale)	
Follow-up and Outcomes	10a	Clinician and patient-assessed outcomes (if available)	125-127
	10b	Important follow-up diagnostic and other test results	127-131
	10c	Intervention adherence and tolerability (How was this assessed?)	130-131
	10d	Adverse and unanticipated events	
Discussion	11a	A scientific discussion of the strengths AND limitations associated with this case report	126-139
	11b	Discussion of the relevant medical literature **with references**.	151-178
	11c	The scientific rationale for any conclusions (including assessment of possible causes)	178-184
	11d	The primary “take-away” lessons of this case report (without references) in a one paragraph conclusion	188-191
Patient Perspective	12	The patient should share their perspective in one to two paragraphs on the treatment(s) they received	
Informed Consent	13	Did the patient give informed consent? Please provide if requested	Yes ☑ No ☐
